# Infective Spondylitis with Epidural Abscess Formation Caused by *Roseomonas mucosa*: A Case Report and Literature Review

**DOI:** 10.1155/2023/6332814

**Published:** 2023-05-22

**Authors:** Zong-Han Lin, Yu-Chang Lu, Kuan-Sheng Wu

**Affiliations:** ^1^Department of Medical Education and Research, Kaohsiung Veterans General Hospital, Kaohsiung, Taiwan; ^2^Division of Infectious Diseases, Department of Internal Medicine, Kaohsiung Veterans General Hospital, Kaohsiung, Taiwan; ^3^School of Medicine, National Yang Ming Chiao Tung University, Taipei, Taiwan

## Abstract

*Roseomonas mucosa* (*R. mucosa*) is a pink-pigmented, aerobic, nonfermentative, slow-growing Gram-negative coccus typically isolated from the natural environment, human skin, and hospital environment. This pathogen, in most circumstances, leads to infections in immunocompromised hosts, but it may sometimes invade immunocompetent individuals. Bacteraemia is the most common form of infection caused by *R. mucosa*. In contrast, only two case reports have described *R. mucosa*-related epidural abscess formation and infective spondylitis. In this case report, we shared the history and treatment experience of a 76-year-old female who was diagnosed with infective spondylitis and epidural abscess caused by *R. mucosa*. She received a local transdermal injection into the lower back to relieve her back pain two months before symptom onset, which was considered to be associated with this infection episode. After admission to the hospital, neurosurgeons performed emergent decompression and debridement. She was treated with intravenous ceftriaxone for four weeks, followed by oral ciprofloxacin for another eight weeks. The patient recovered well without any sequelae and had no relapse of infection at least six months after the end of treatment. In addition to the case report, we reviewed the literature for reported cases caused by *R. mucosa*. Our experience suggests that clinicians should include *R. mucosa* as one of the possible healthcare-associated pathogens among individuals who have undergone transdermal procedures. We believe that this article will help clinicians better recognize *R. mucosa* infection.

## 1. Introduction

The *Roseomonas* species was first documented in 1993 [1], and in 2003, *R. mucosa* was established as a separate species from *Roseomonas gilardii* because of its distinct genotype and phenotype [2]. This Gram-negative coccus has been isolated from the natural environment [3–6], human skin [7], and hospital environment [8]. It is generally believed that people infected with *R. mucosa* are primarily immunocompromised [9] or have underlying diseases [10]. However, a few reports have revealed that *R. mucosa* also infects immunocompetent individuals and might lead to severe infections [11].

Epidural abscess is a severe central nervous system infection [12] and may lead to fever, back pain, neurological deficits, impaired motor function, paralysis, and even death [13]. *R. mucosa* is an infrequent cause of epidural abscess formation. In this case report, we present the disease course of a female diagnosed with infective spondylitis and epidural abscess formation caused by *R. mucosa*. In addition, we reviewed the literature and listed all published articles on *R. mucosa* infection.

## 2. Case Presentation

### 2.1. Clinical Course

A 76-year-old female with independent daily activity presented with progressive low back pain for five days and fever for three days. She had a medical history of type 2 diabetes mellitus under reasonable medical control and left breast cancer, for which she had undergone surgical resection five years prior.

Two years prior, she was diagnosed with spinal retrolisthesis over L2-L5. In addition, an orthopaedist noted a collapsed disc at L3-L4 with bilateral nerve root compression. She had been experiencing intermittent low back pain and numbness over the left leg since that time and sought rehabilitation, taking painkillers as needed. Orthopaedics did not suggest surgical intervention in consideration of her severe osteoporosis and independent daily activity status. To relieve the low back pain, the patient sought various treatment modalities over the past two years. Two months before admission, she underwent a so-called “nerve block” procedure by receiving a local transdermal injection into her lower back. One month prior, she received radiofrequency coagulation pain therapy at a local hospital.

Her low back pain became more severe and unbearable five days before this admission. She fell at home three days prior and was admitted to a local hospital. Her body temperature was 39.0°C upon admission, with otherwise typical vital signs. Because of the severe painful disability, she could no longer stand and laid in bed all day. She was also put on a Foley catheter due to difficulty in urination. Under the suspicion of infective spondylitis, contrast L-spine magnetic resonance imaging (MRI) was performed and revealed an L3-L4 collapsed disc with nerve compression, which was not significantly different from previous findings. Having persistent fever and progressive low back pain, she was transferred to Kaohsiung Veterans General Hospital (KVGH), a tertiary teaching hospital, for further management.

**Figure 1:**
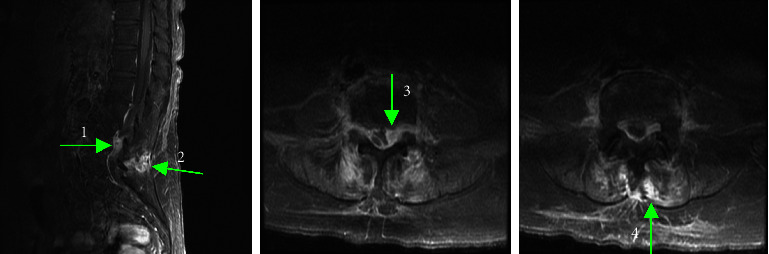
T1 sagittal and axial contrast-enhanced MRI obtained prior to surgery, showing focal, small, lobulated fluid collection at the posterior epidural space of L4-L5 (arrows 1 and 3), with adjacent soft-tissue swelling at the epidural space and posterior column of the L4-L5 level (arrows 2 and 4).

Upon admission to KVGH, her vital signs were as follows: blood pressure 132/63 mmHg, pulse rate 74/min, respiratory rate 17/min, body temperature 38.4°C, and oxygen saturation 97%. On physical examination, her consciousness was clear, and there was a marked tender point over her lower back. The muscle power over the bilateral lower limbs was symmetrically decreased (grade 2 out of 5), while the muscle power over the bilateral upper limbs was intact. A bilateral straight leg raise test was positive, and the deep tendon reflex over the bilateral lower limbs was normal. Laboratory data reported leukocytosis, elevated erythrocyte sedimentation rate and C-reactive protein (15.38 mg/dl). Urine analysis showed pyuria, and urine culture yielded no pathogen. Under a high suspicion of infective spondylitis with/without epidural abscess formation, cefepime and vancomycin were given as empirical antibiotics and an MRI was repeated on hospital Day 3. The images showed a focal, small, lobulated fluid collection at the posterior epidural space of L4-L5, with adjacent soft tissue swelling at the epidural space and posterior column of the L4-L5 level. Infective arthritis over the L4-L5 facet joint with adjacent epidural abscess formation leading to severe spinal stenosis at the L4-L5 level was concluded by the radiologist (Figure 1).

Emergent partial laminectomy of L3-L4 and internal decompression of L4-L5 was therefore conducted for debridement and decompression of the lesions. Some dirty fluid and granulation tissue collected during the surgery later yielded *R. mucosa*, which was identified by matrix-assisted laser desorption/ionization time of flight mass spectrometry (MALDI-TOF MS). Both fungal and mycobacterial cultures of the lesions were negative. An antimicrobial sensitivity test of *R. mucosa* was conducted by the E-test method and showed susceptibility to imipenem (0.5 *µ*g/ml), ceftriaxone (1.5 *µ*g/ml), amikacin (0.5 *µ*g/ml), and ciprofloxacin (0.125 *µ*g/ml). The pathological report described a bony tissue with acute and chronic inflammatory cell infiltration, bone necrosis, and focal granulation tissue, which is compatible with osteomyelitis.

**Figure 2:**
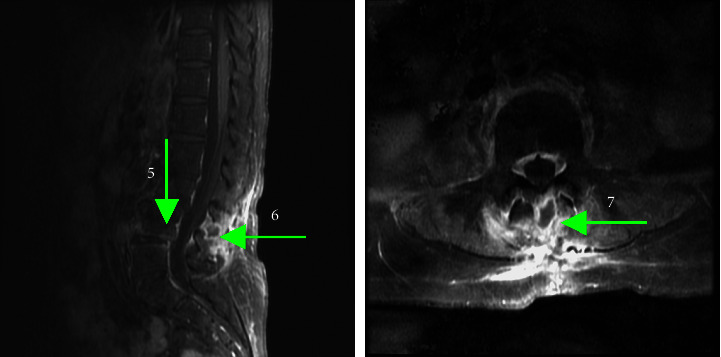
T1 sagittal and axial water-suppression contrast-enhanced MRI obtained approximately 1 month after surgery, showing early stage of infective spondylodiscitis between L4 and L5 (arrow 5) and Baastrup's disease between L3 and L4 with secondary abscess transformation (arrows 6 and 7).

After the spinal surgery, there was a notable improvement in the patient's muscle strength. We utilized the Medical Research Council scale to assess the patient's muscle power, which scores muscle strength from 0 to 5, with 0 indicating no contraction, 1 indicating trace contraction, 2 indicating active movement with gravity eliminated, 3 indicating active movement against gravity, 4 indicating active movement against resistance, and 5 indicating normal power. The patient's muscle power in both lower extremities increased from grade 2 to 4 within two days following the surgery. However, during a follow-up MRI, an abscess measuring 1.5 cm in diameter was discovered at the L3-L4 level. In addition, an early stage of infective spondylodiscitis was noted at L4-L5 (Figure 2). Sonography-guided aspiration of the abscess was attempted but failed due to the small size of the lesion. We treated the patient with intravenous ceftriaxone 2 gm q12 h for four weeks during the hospital course.

Upon discharge from the hospital, her muscle power was grade 4 over the bilateral lower limbs. She could walk under the assistance of a four-foot walker but could not stand up by herself. The patient received another two months of oral antibiotics of ciprofloxacin 500 mg PO q12 h at the outpatient department. She recovered three months after the infection. Her muscle power in both lower limbs was graded as 5. She was able to walk with the assistance of a four-foot walker and could stand up independently. Although she still experienced some soreness in her left leg, there was no mention of any low back pain. There was no relapse of infection at least six months after the end of treatment.

### 2.2. Laboratory Method

The laboratory of KVGH inoculated the epidural abscess specimen onto blood agar, eosin methylene blue (EMB) agar, chocolate agar, Columbia C.N.A. agar, and thioglycolate broth. The following day, pink colonies were observed on EMB agar. After subsequent subculture and identification using MALDI-TOF MS, the colonies were identified as *Roseomonas mucosa*. Turbidity was also observed in the thioglycolate broth the following day, and subsequent subculture and identification yielded the same result of *Roseomonas mucosa*.

The KVGH laboratory routinely uses VITEK MS MALDI-TOF (BioMérieux, France) to identify bacterial species. The process for identifying bacteria using MALDI-TOF MS is as follows. First, a toothpick is used to pick up a colony from the agar plate and spread it evenly onto the center of a sample plate to form a thin bacterial film. Then, 1 *μ*l of VITEK MS-CHCA matrix solution is pipetted on the bacterial film, allowed to dry and then proceeded for identification. The strain of *Roseomonas mucosa* was identified using MALDI-TOF MS three times, with consistent results indicating a single bacterium and a high confidence value of 99.9.

The KVGH laboratory routinely uses the VITEK 2 system (BioMérieux, France) for antibiotic susceptibility testing. However, in the case of this *Roseomonas mucosa*, VITEK 2 system failed to generate the antibiotic susceptibility testing result. Therefore, the E-test method was used instead, with the following procedure: first, 1-2 colonies were picked up from the agar using a sterile cotton swab and suspended in tryptic soy broth adjusted to McFarland No. 0.5 turbidity. Then, the swab was used to streak the Mueller–Hinton agar in three directions and allowed to dry for 2-3 minutes. The E-test strip was then carefully placed on the agar surface and incubated in a non-CO_2_ 35°C ± 2°C incubator for 16–20 hours before interpreting. Antimicrobial susceptibility was interpreted according to the latest Clinical and Laboratory Standards Institute Guideline (M100-S 31).

## 3. Discussion

In the past, it was generally believed that *R. mucosa* is not highly pathogenic to immunocompetent populations. A systematic review [9] suggests that *R. mucosa* infections occur more frequently in immunocompromised patients or patients with severe comorbidities, including dialysis [14, 15], cancer [16, 17], HIV [18], and autoimmune disease [10]. In addition, the placement of catheter should be considered a risk factor [19, 20], and the assessment of whether to remove the relevant catheter after infection is considered an essential part of the treatment [21]. However, there is a growing body of the literature reporting that *R. mucosa* can potentially cause severe infections in immunocompetent patients [11, 22]. The most common infection caused by *R. mucosa* is bacteraemia. Other types of infections, although uncommon, have been reported sporadically (Table 1). We searched PubMed using the keyword combinations “*Roseomonas*,” “*Roseomonas mucosa*,” “epidural abscess,” and “infective spondylitis.” Only one case report described an epidural abscess due to *R. mucosa* infection [27]; another article reported a male with infective spondylitis caused by *R. mucosa* infection [11]. To the best of our knowledge, this article is the second reported case of infective spondylitis and epidural abscess caused by *R. mucosa* in the literature.

According to previous reports, *R. mucosa* is usually resistant to penicillins and cephalosporins and susceptible to carbapenems, tetracyclines, fluoroquinolones, and aminoglycosides [9, 35]. Our drug susceptibility tests of the *R. mucosa* isolate were similar to the literature. We treated the patient with intravenous ceftriaxone for four weeks, followed by oral ciprofloxacin for another eight weeks. The outcome was good, and there was no relapse of infection at least six months after the end of treatment.

*R. mucosa* is an easily misidentified species. A previous study [36] compared the identification ability of three routine laboratory bacterial identification methods for *R. mucosa*, including VITEK 2, MALDI-TOF MS, and 16S rRNA gene sequencing. The VITEK 2 system could misidentify the isolate as *Rhizobium radiobacter*, *Sphingomonas paucimobilis*, or *R. gilardii* [10, 27]. In comparison, MALDI-TOF MS and 16S rRNA gene sequencing had a more reliable identification ability. Generally speaking, 16S rRNA sequencing is often considered a superior method to MALDI-TOF MS for bacterial identification, as it provides greater taxonomic resolution at the species level. However, MALDI-TOF MS can also provide accurate identification of bacteria by comparing the mass-to-charge ratio and the mass spectra obtained by measuring the time of the bacteria proteins, which have been laser ionized, flight to the detector with those in a reference database [37]. The main reason for using MALDI-TOF in this current report is that it is the routine method in the KVGH laboratory and is most relevant to clinical practice. After all, 16S rRNA sequencing is mostly used for research purposes rather than clinical applications. In addition, MALDI-TOF is relatively inexpensive, has a higher accessibility, and can provide results within a few hours.

After compiling much of the literature on the subject, we have summarized the valuable lessons learned from this case and our treatment advice for similar cases. First, according to previous reports, *R. mucos*a is present in human skin [7] and medical settings [8]. A previous report [27] described that invasive interventions might cause skeletal muscle infections caused by the *Roseomonas* species. Our patient underwent several medical procedures before the onset of infection, including nerve block with local lidocaine injection and radiofrequency coagulation. We suspected that this infection episode was associated with one of the medical procedures, especially nerve block injection. Therefore, we suggest that among individuals who have healthcare-associated infections, especially those who have undergone transdermal procedures, clinicians should include *R. mucosa* as one of the possible pathogens [8]. Second, to identify the type of microorganism infected, we recommend using MALDI-TOF MS or 16S rRNA gene sequencing to avoid misidentifying strains. Finally, for the treatment, we recommend that once the location of the infection is identified, treatment should prioritize the removal of the source of the infection. Besides, since the antibiotics recommended by the Infectious Diseases Society of America for the prevention of postsurgical skin and soft tissue infections are usually not effective against *R. mucosa* [38], we recommend the use of carbapenems, tetracycline, fluoroquinolone, or aminoglycosides when *R. mucosa* infection is identified, as *R. mucosa* is usually sensitive to these antibiotics.

There are two major limitations to this report. The first limitation is related to the method of bacterial identification utilized. Instead of using the gold standard of 16S rRNA sequencing, we relied on the clinically common MALDI-TOF MS method. While MALDI-TOF MS is commonly used in clinical settings and provides rapid results, it may not provide the same level of taxonomic resolution as 16S rRNA sequencing. The second limitation is that although we have reviewed most of the literature on the diseases related to *R. mucosa*, we only encountered one actual case, and our clinical experience with this species is still insufficient.

In conclusion, although rare, *R. mucosa* can cause epidural abscess formation and infective spondylitis in immunocompetent hosts. Since *R. mucosa* has fewer antibiotic choices, clinicians should arrange surgical removal or drainage as soon as possible once abscess formation occurs. In addition, *R. mucosa* should be considered a pathogen associated with medical procedures.

## Figures and Tables

**Table 1:** Summary of reported infection cases caused by *Roseomonas mucosa* in the literature.

Reference	Age/sex	Infection	Underlying disease/risk factors	Identification	Drug resistance	Treatment	Outcome
Dé et al., 2004 [23]	21/F	Bacteraemia	Leukaemia	16S rRNA gene sequencing	n.d.	Removed CVC IV LFX ⟶ oral LFX + AMC IV IPM + VCM	Resolved
Dé et al., 2004 [23]	65/M	Bacteraemia	Lymphoma	16S rRNA gene sequencing	n.d.	Various antibiotics	Resolved
Dé et al., 2004 [23]	54/M	Bacteraemia	Leukaemia	16S rRNA gene sequencing	n.d.	IPM + VCM ⟶ FEP + VCM	Resolved
Christakis et al., 2006 [16]	39/M	Bacteraemia	Acute myelogenous leukaemia with neutropenic state	Identified as *Roseomonas* sp. by RapidID NF PLUS Misidentified as Methylobacterium mesophilicum by RapidID NF PLUS Identified as *R. mucosa* by 16S rRNA gene sequencing	R: CAZ, AZT, CTX, TZP, TCC, and TMP-SMX S: CFX, GM, TM, IPM, MEM, LFX, gatifloxacin, TE, AMK, FEP, and C	AMK + TZP for 10 Ds	Resolved
Elshibly et al., 2005 [24]	42/M	Bacteraemia	Diffuse large B-cell non-Hodgkin's lymphoma	16S rRNA gene sequencing	R: ABPC, AZT, CTX, CAZ, CXM, CFX, AMC, MEM, TZP, TMP, and IPM S: AMK, colomycin, GM, NET, and TM	TZP + GM ⟶ TEC + TZP + GM	Resolved
Sipsas et al., 2006 [25]	40/M	Septic arthritis	Rheumatoid arthritis receiving infliximab therapy	16S rRNA gene sequencing	R: benzylpenicillin, TZP, FOX, CRO, and CAZ S: CM, IPM, TE, DO, CFX, AMK, and GM	D 1–3: IV CRO D 4–60: oral CFX	Resolved
Bard et al., 2010 [26]	18/M	Bacteraemia	Multiple episodes of urinary tract infection and bacteraemia	16S rRNA gene sequencing	R: ABPC, CEZ, CAZ, CRO, TZP, and TMP-SMX S: AMK, CFX, GM, IPM, LFX, MEM, and TM	VCM + CRO ⟶ CFX for 10 Ds	Resolved
Boyd et al., 2012 [18]	19/M	Peritonitis	HIV + ESRD with PD	Misidentified as Ochrobactrum anthropi and Acinetobacter lwoffii by the VITEK 2 automated system Misidentified as Methylobacterium mesophilicum by the API 20 NE system Identified as *R. mucosa* by 16S rRNA gene sequencing	R: TZP, CAZ, and TMP-SMX S: AMK, CRO, CFX, LFX, GM, IPM, MEM, and FEP	D 1–14: IP/IV CAZ + VCM D 14–35: IP CFX + GM + VCM D 35–49: IP CRO	PD failed; long-term haemodialysis commenced
Maraki et al., 2013 [27]	54/F	Epidural abscess	Instrumented posterior lumbar interbody fusion	Misidentified as *R. gilardii* by the VITEK 2 automated system Identified as *R. mucosa* by 16S rRNA gene sequencing	R: beta-lactams (except CTX), FO, and colistin S: carbapenems, aminoglycosides, fluoroquinolones, TEs, TGC, C, MNO, and TMP-SMX	Surgical debridement D 1–24: IV VCM + oral rifampin D 25–81: MEM	Resolved
Al-Anazi et al., 2013 [28]	41/F	Bacteraemia	T-lymphoblastic lymphoma	Identified as *Roseomonas* by the VITEK 2 and the Microseq 500 16S rDNA bacterial identification kit Identified as *R. mucosa* by 16S rRNA gene sequencing	R: PIP, TZP, and all cephalosporins S: IPM, GM, AMK, and CFX	Removed CVC D 1–4: IV TZP D 4–14: IV IPM + AMK CFX	Resolved
Michon et al., 2014 [17]	3/n.d.	Bacteraemia	B-lineage acute lymphoblastic leukaemia	16S rRNA gene sequencing	R: TZP and IPM S: AMK	D 1–4: TZP D 4–7: IPM + AMK D 7–14: oral CFM	Resolved
Matsukuma et al., 2014 [14]	61/M	Peritonitis	Diabetic nephropathy and receiving PD for ESRD	16S rRNA gene sequencing	R: cephalosporins, including CAZ S: GM, CFX and MEM	D 1–3: IP CEZ + AMK D 4–6: IP CAZ D 7–28: IP CFX	Resolved, changed to haemodialysis
Kim et al., 2015 [11]	74/M	Infective spondylitis with bacteraemia	Vertebroplasty for compression fractures and laminectomy for spinal stenosis	Misidentified as *R. gilardii* by the VITEK 2 automated system Identified as *R. mucosa* by 16S rRNA gene sequencing	R: CFX, GM, and PIP S: carbapenems, CAZ, TIC, and TM	D 1–7: IV CEZ D 8–15: CAZ 1 month later: received total corpectomy + IV CAZ for 6 weeks	Resolved
Kim et al., 2016 [29]	84/F	Bacteraemia	Common bile duct obstruction	Misidentified as *R. gilardii* by the VITEK 2 automated system Identified as *R. mucosa* by 16S rRNA gene sequencing	R: ABPC and CAZ S: aminoglycosides, carbapenems, fluoroquinolones, and CTX	CTX + MET	Resolved
Kim et al., 2016 [29]	17/M	Bacteraemia	Acute myeloid leukaemia	Misidentified as *R. gilardii* by the VITEK 2 automated system Identified as *R. mucosa* by 16S rRNA gene sequencing	R: ABPC and CAZ S: aminoglycosides, carbapenems, fluoroquinolones, and CTX	CAZ + aminoglycoside ⟶ VCM + carbapenem	Resolved
Bhende et al., 2017 [30]	74/M	Endogenous endophthalmitis	DM	16S rRNA gene sequencing	n.d.	D 1: intravitreal injection of CAZ + oral CTX + topical moxifloxacin + TM D 2: intravitreal injection of VCM + CAZ Received vitreous surgery + intravitreal CAZ Postoperation: oral CFX + moxifloxacin + TM drops for 5 Ds	Resolved
Abu Choudhury et al., 2017 [19]	53/M	Bacteraemia	Peripheral intravenous catheter insertion	Illumina Nextera XT and sequenced via the Illumina HiSeq 2000	n.d.	Cephalosporin	No systemic infection
Diesendorf et al., 2017 [31]	n.d.	Acute pulpitis	n.d.	MALDI-TOF mass spectrometry	R: ABPC, ABPC/SBT, PIP, TZP CEZ, CXM, CAZ, and FO S: CRO, IPM, MEM, GM, TM, AMK CFX, TMP-SMX, TE, TGC, and polymyxin B	n.d.	n.d.
Kimura et al., 2018 [32]	9/M	Bacteraemia	Febrile neutropenia caused by chemotherapy for cerebellar medulloblastoma	16S rDNA sequencing	R: PIP, AZT, and CAZ S: carbapenems, aminoglycosides, MNO, fluoroquinolones, and TMP-SMX	MEM	Resolved
Shao et al., 2019 [10]	44/F	Infective endocarditis	Systemic lupus erythematosus	Misidentified as *R. gilardii* by the VITEK 2 automated system Identified as *R. mucosa* by 16S rRNA gene sequencing	R: TZP, ABPC, CAZ, FEP, and colistin S: AMK, GM, LFX, CFX, IPM, and MEM	D 4–7: VCM + TZP D 8–11: TZP + LFX D 12–25: MEM + AMK	Resolved
Beucler et al., 2020 [33]	27/M	Peritonitis	VPS ventricular catheter for congenital hydrocephalus	MALDI-TOF mass spectrometry	R: TIC, TZP, CAZ, and FO S: IPM, MEM, GM, AMK, and CFX	Removal of the shunt D 1–9: CTX + linezolid	Resolved
Waris et al., 2021 [22]	17/M	Meningitis	Left temporal craniotomy for open fenestration of an arachnoid cyst	Misidentified as *R. gilardii* by the VITEK 2 automated system Identified as *R. mucosa* by 16S rRNA gene sequencing	S: LFX and MEM	D 1-2: IV MEM D 2–8: oral LFX D 10–12: IV MEM + VCM D 12–47: IV MEM	Resolved
Roy et al., 2021 [15]	65/F	Peritonitis	PD for ESRD	MALDI-TOF mass spectrometry	R: cephalosporin S: GM	Removed the PD catheter IP GM for 3 weeks	Resolved
Spindel et al., 2022 [34]	30/M	Pyogenic hepatic abscess with cardiac tamponade	Daily tobacco and marijuana smoking	MALDI-TOF mass spectrometry	S: CRO and CFX	Emergent pericardiocentesis + hepatic and pericardial drains for 12 days D 1–5: TZP + MET D 5–40: CRO + MET	Resolved Metastatic hepatoid carcinoma noted 1 year later
Our case	76/F	Infective spondylitis with epidural abscess	Nerve block with local anaesthesia and radiofrequency coagulation in the lumbar spine	MALDI-TOF mass spectrometry	S: IPM, CRO, AMK, and CFX	Partial laminectomy + debridement D 1–7: FEP + VCM D 5: Debridement D 8–39: CRO D 39–99: CFX	Resolved

ABPC: ampicillin; AMC: amoxicillin-clavulanic acid; AMK: amikacin; AZT: aztreonam; C: chloramphenicol; CAZ: ceftazidime; CEZ: cefazolin; CFM: cefixime; CM: clindamycin; CRO: ceftriaxone; CFX: ciprofloxacin; CTX: cefotaxime; CXM: cefuroxime; D: day; DM: diabetes mellitus; DO: doxycycline; ESRD: end-stage renal disease; FEP: cefepime; FO: fosfomycin; FOX: cefoxitin; GM: gentamicin; IP: intraperitoneal; IPM: imipenem; IV: intravenous; LFX: levofloxacin; MEM: meropenem; MET: metronidazole; MNO: minocycline; n.d.: not described; NET: netilmicin; PD: peritoneal dialysis; PIP: piperacillin; R: resistant; *R. mucosa*: *Roseomonas mucosa*; *R. gilardii*: *Roseomonas gilardii*; S: susceptible; SBT: sulbactam; TCC: ticarcillin-clavulanic acid; TEC: teicoplanin; TE: tetracycline; TGC: tigecycline; TIC: ticarcillin; TM: tobramycin; TMP-SMX: trimethoprim-sulfamethoxazole; TZP: piperacillin-tazobactam; VCM: vancomycin.

## Data Availability

The health record data used to support the findings of this case report are restricted to ensure patient privacy. Specific health record data have already been included within the article in a manner that does not compromise patient confidentiality.
